# Phosphate group functionalized magnetic metal–organic framework nanocomposite for highly efficient removal of U(VI) from aqueous solution

**DOI:** 10.1038/s41598-021-03246-3

**Published:** 2021-12-21

**Authors:** Changfen Bi, Baoxin Zheng, Ye Yuan, Hongxin Ning, Wenfeng Gou, Jianghong Guo, Langxing Chen, Wenbin Hou, Yiliang Li

**Affiliations:** 1 Tianjin Key Laboratory of Radiation Medicine and Molecular Nuclear Medicine, Institute of Radiation Medicine, Peking Union Medical College, Chinese Academy of Medical Sciences, Tianjin, 300192 People’s Republic of China; 2 College of Traditional Chinese Medicine, Tianjin University of Traditional Chinese Medicine, Tianjin, 301617 People’s Republic of China; 3 Tianjin Key Laboratory of Biosensing and Molecular Recognition, State Key Laboratory of Medicinal Chemical Biology, Research Center for Analytical Sciences, College of Chemistry, Nankai University, Tianjin, 300071 People’s Republic of China

**Keywords:** Environmental sciences, Materials science

## Abstract

The phosphate group functionalized metal-organic frameworks (MOFs) as the adsorbent for removal of U(VI) from aqueous solution still suffer from low adsorption efficiency, due to the low grafting rate of groups into the skeleton structure. Herein, a novel phosphate group functionalized metal–organic framework nanoparticles (denoted as Fe_3_O_4_@SiO_2_@UiO-66-TPP NPs) designed and prepared by the chelation between Zr and phytic acid, showing fast adsorption rate and outstanding selectivity in aqueous media including 10 coexisting ions. The Fe_3_O_4_@SiO_2_@UiO-66-TPP was properly characterized by TEM, FT-IR, BET, VSM and Zeta potential measurement. The removal performance of Fe_3_O_4_@SiO_2_@UiO-66-TPP for U(VI) was investigated systematically using batch experiments under different conditions, including solution pH, incubation time, temperature and initial U(VI) concentration. The adsorption kinetics, isotherm, selectivity studies revealed that Fe_3_O_4_@SiO_2_@UiO-66-TPP NPs possess fast adsorption rates (approximately 15 min to reach equilibrium), high adsorption capacities (307.8 mg/g) and outstanding selectivity (*S*_u_ = 94.4%) towards U(VI), which in terms of performance are much better than most of the other magnetic adsorbents. Furthermore, the adsorbent could be reused for U(VI) removal without obvious loss of adsorption capacity after five consecutive cycles. The research work provides a novel strategy to assemble phosphate group-functionalized MOFs.

## Introduction

Uranium is not only a sustainable fuel source, but also a chemically toxic and radioactive pollutant for both the ecological environment and human beings^1,2^. Large amounts of uranium-containing waste have been released to natural water in a variety of ways, such as incorrect uranium mining, nuclear fuel fabrication and natural weathering^3–5^. Due to long-term radiation toxicity, uranium can not only cause carcinogenic, teratogenic or mutagenic radiation damage to human organs such as liver, kidney, skin and bone, but also result in a lasting and disastrous impact on the ecological environment^6–8^. Therefore, the removal and recovery of uranium from wastewater is very significant not only for ecological stability and human health, but also for nuclear sustainable development^9,10^.

Several strategies have been developed for radioactive wastewater treatment including coagulation^11^, membrane separation^12,13^, ion-exchange^14,15^, reductive precipitation^16,17^ and adsorption^9,18,19^. Among these strategies, adsorption has been proven to be the most effective treatment method owning to its low cost, simple operation and environment compatibility^20–22^. In the past decades, various adsorption materials such as silica nanospheres, polymeric nanoparticles, carbon-based materials, and advanced porous materials have been extensively developed for U(VI) removal^23–28^. Metal–organic frameworks (MOFs) are a particular class of porous materials constituted by metallic cations/clusters with organic ligands, and have superior intrinsic properties including tunability, crystallinity, stability and chemical versatility^29–31^. And as a result, they has been widely applied in various fields, such as gas storage^32,33^, catalysis^34–38^, drug delivery^39,40^ sensing^41,42^ and separation^43–46^. At present, a variety of MOFs (i. e. ZIF-8, MIL-101, UiO-66) have been already designed and synthesized for U(VI) capture from aqueous solution^47–49^.

Since in many cases of radioactive pollution, the wastewater is acidic, which requires adsorbents to have an adequate stability in acidic aqueous solutions. Based on this consideration, a zirconium-based MOF (UiO-66 and UiO-66-NH_2_) composed of a Zr_6_(µ_3_-O)_4_(µ_3_-OH)_4_ or 12-connected Zr_6_(µ_3_-O)_4_(µ_3_-OH)_4_(NH_2_-COO)_12_ cluster that possess strong Zr–O bonds was selected as a potential adsorbent due to its exceptional stability in acidic solutions^50–55^. Hierarchical porous and functional group (–OH, –NH_2_, –COOH, =N–OH) post-modified UiO-66 have been prepared and studied in rapid U(VI) removal from an aqueous solution^53–58^. It is also a wise strategy to modify phosphate groups on MOFs, due to strong coordination ability of phosphate groups with U(VI)^59–61^. However, owning to the low grafting rate of phosphate groups into the skeleton structure, reported MOFs dotted with the phosphate group (UiO-68-P(O)(OEt)_2_, UiO-68-P(O)(OH)_2_, MIL-101-ship, Zr_7_P_8_) have low adsorption efficiency towards U(VI), which seriously affect their further application^62–64^.

Phytic acid, as myoinositol hexaphosphate extracted from plants, has strong complexation ability to metal ions^65–67^. In this study, we developed a phosphate group functionalized metal–organic framework nanoparticle by the chelation between Zr(IV) and phytic acid, and loaded Fe_3_O_4_ nanoparticles to achieve fast magnetic separation of U(VI) from an aqueous solution (denoted as Fe_3_O_4_@SiO_2_@UiO-66-TPP). The nanoparticle was synthesized by simple steps and properly characterized by transmission electron microscopy (TEM), Fourier transform infrared (FT-IR) spectroscopy, Brunauer–Emmett–Teller (BET) measurements and vibrating sample magnetometer (VSM). The removal performance of Fe_3_O_4_@SiO_2_@UiO-66-TPP for U(VI) was evaluated using batch experiments under different adsorption conditions, including solution pH, incubation time, temperature and initial U(VI) concentration. Fortunately, the nanoparticle possessed fast adsorption rates (approximately 15 min to reach equilibrium), high adsorption capacity (307.8 mg/g) and selectivity (94.4%) towards U(VI). Finally, the adsorption dynamics, isotherms and mechanism of Fe_3_O_4_@SiO_2_@UiO-66-TPP for U(VI) were also discussed.

## Experimental section

### Materials

Cobaltous nitrate hexahydrate (Co(NO_3_)_2_·6H_2_O), gadolinium nitrate hexahydrate (Gd(NO_3_)_3_·6H_2_O), lanthanum nitrate hexahydrate (La(NO_3_)_3_·6H_2_O), neodymium nitrate hexahydrate (Nd(NO_3_)_3_·6H_2_O), ytterbium nitrate pentahydrate (Yb(NO_3_)_3_·5H_2_O) and samarium nitrate hexahydrate (Sm(NO_3_)_3_·6H_2_O) were obtained from Sigma-Aldrich (USA). Nickel nitrate hexahydrate (Ni(NO_3_)_2_·6H_2_O), strontium nitrate (Sr(NO_3_)_2_) and zinc nitrate hexahydrate (Zn(NO_3_)_2_·6H_2_O) were obtained from Damao Chemical Reagent Factory (Tianjin, China). Uranium nitrate oxide (UO_2_(NO_3_)_2_) was obtained from Chemical Reagent Purchasing and Supply Station (Shanghai, China). Zirconium chloride (ZrCl_4_)) and 2-aminoterephthalic acid were obtained from Heowns Biochemical Technology Co., Ltd (Tianjin, China). Phytic acid sodium salt were purchased fromSolarbio Science & Technology Co., Ltd (Beijing, China). Tetraethoxysilane (TEOS) was obtained from J&K Chemicals Ltd (China). Iron (III) chloride hexahydrate (FeCl_3_·6H_2_O), Sulfuric acid (H_2_SO_4_), nitric acid (HNO_3_), hydrochloric acid (HCl), ethylene glycol (EG), N, N-dimethylformamide (DMF), methanol (MeOH), ethanol (EtOH), and other reagents were obtained from Tianjin Chemical Reagent No. 6 Factory (China). High-purity deionized water (18.2 MΩ cm) was obtained from a Millipore Milli-Q direct water purification system (USA).

### Preparation of silica layer coated magnetic nanoparticles (Fe_3_O_4_@SiO_2_)

Naked Fe_3_O_4_ NPs were prepared by a the facile solvothermal method. Typically, 2.70 g FeCl_3_·6H_2_O, 7.71 g ammonium acetate, 0.8 g sodium citrate were dissolved in 140 mL EG solution under sonication to form a homogenous solution. Then the mixture was heated at 200 °C for 16 h after transferring to a Teflon-lined stainless-steel autoclave (200 mL). Under an external magnetic field, the prepared Fe_3_O_4_ NPs were separated from the reaction solvent, washed with water and EtOH for several times in turn, and dried in vacuum at 40 °C.

300 mg Fe_3_O_4_ NPs were dispersed in the mixed solution of 0.75 mL ammonium hydroxide, 12 mL water and 46 mL EtOH under sonication, and then 0.9 mL TEOS in 3 mL EtOH was added dropwise to the above mixed solution. The mixture was stirred magnetically for 12 h at room temperature. Under an external magnetic field, the prepared Fe_3_O_4_@SiO_2_ NPs were separated from the reaction solvent and washed with water and EtOH for several times in turn, and dried in vacuum at 40 °C.

### Preparation of magnetic UiO_2_-66-NH_2_ (Fe_3_O_4_@SiO_2_@UiO_2_-66-NH_2_)

200 mg Fe_3_O_4_@SiO_2_ NPs and 466 mg ZrCl_4_ were dispersed in 30 mL DMF under sonication for 30 min, and then 362 mg dissolved in 30 mL DMF was added to the solution. The mixture was heated at 120 °C for 6 h. Under an external magnetic field, the prepared Fe_3_O_4_@SiO_2_@UiO_2_-66-NH_2_ NPs were separated from the reaction solvent and washed with DMF and MeOH, immersed in MeOH for 3 d, and finally dried in vacuum at 40 °C.

### Preparation of phosphate group functionalized magnetic metal–organic frameworks nanoparticles (Fe_3_O_4_@SiO_2_@UiO_2_-66-TPP)

15 mg phytic acid sodium salt was dissolved in the mixed solution of 4 mL water and 20 mL CH_3_COOH (2%, v/v), and then 40 mg Fe_3_O_4_@SiO_2_@UiO-66-NH_2_ was dispersed in the above solution under sonication. After pH value of the reaction system was adjusted to 5, the mixture was heated at 50 °C for 30 min, and then kept at 115 °C for 12 h. Under an external magnetic field, the prepared Fe_3_O_4_@SiO_2_@UiO_2_-66-TPP NPs were separated from the reaction solvent and washed with water to neutral, and dried in vacuum at 40 °C.

## Characterizations

The synthesized nanoparticles were systematically characterized through diverse techniques including transmission electron microscopy (TEM), Fourier transform infrared spectroscopy (FT-IR) and vibration sample magnetometer (VSM). The specific surface area, total pore volume, pore size and the Zeta potentials were also measured.Transmission electron microscopy (TEM) and composition mapping images were taken with a JEM-2100 (Japan) transmission electron microscope. Fourier transform infrared (FT-IR) spectra (4000–400 cm^−1^) were obtained on a BRUKER TENSOR 27 (Germany) Fourier transform infrared spectrophotometer in KBr pellets. The N_2_ adsorption–desorption isotherms were analyzed on a Micromeritics ASAP (USA) 2010 apparatus. The magnetic properties were characterized using a LDJ9600-1 (USA) vibrating sample magnetometer (VSM). The zeta potentials under different pH conditions were measured with a Brookhaven ZetaPALS (USA) analyzer at room temperature.

### Batch experiments

To evaluate the performance of the magnetic adsorbents for the removal of U(VI) from aqueous solution, batch sorption experiments were carried out in 45 mL polyethylene tubes. Briefly, 10 mg magnetic adsorbents were dispersed in 25 mL uranium solution or multi-ion solution at a certain pH value adjusted by adding negligible volume of dilute HCl or NaOH solution. After being incubated for a given time at a certain temperature, the magnetic nanocomposites loaded with U(VI) were separated under an external magnetic field. The concentration of UO_2_^2+^ in the supernatant was calculated by determining absorption of its complex with arsenazo(III) at 656 nm using an ultraviolet–visible spectrometer (SHIMADZU UV-1750). The concentration of metal ions including U(VI) was analyzed by inductively coupled plasma mass spectrometry (ICP-MS), when investigating the effect of coexisting ions and ionic strength on adsorption capacity of the magnetic adsorbent for U(VI). The adsorption capacity (q_e_ (mg/g)) and adsorption percentage (% adsorption) were calculated according to the following two equations:

1 $${\text{q}}_{{\text{e}}} = \frac{{\left( {{\text{C}}_{0} - {\text{C}}_{{\text{e}}} } \right) \times {\text{V}}}}{{\text{m}}}$$

2 $$\% \;{\text{adsorption}} = \frac{{{\text{C}}_{0} - {\text{C}}_{{\text{e}}} }}{{{\text{C}}_{0} }} \times 100$$

where C_0_ and C_e_ (mg/L) are the initial and equilibrium concentrations of UO_2_^2+^ in the solution, respectively; V (L) is the volume of solution; and m (g) is the weight of the magnetic adsorbents.

Uranium-selectivity (*S*_u_) to reflect the level of adsorption selectivity of the magnetic adsorbent towards U(VI), which was calculated according to the following equation:

3 $$S_{{\text{u}}} = \frac{{{\text{q}}_{{{\text{e}}({\text{U}})}} }}{{{\text{q}}_{{{\text{e}}({\text{tol}})}} }} \times 100\%$$

where q_e(U)_ and q_e(tol)_ are the U(VI) sorption capacity and all metal ions including U(VI) for the magnetic adsorbent, respectively.

For acquisition of the optimal adsorption performance of Fe_3_O_4_@SiO_2_@UiO-66-TPP NPs towards U(VI), the influence factors, including pH (1.5–5.5), contact time (1–180 min), C_0_ (20–400 mg/L), temperature (298–318 K), and ionic strength were also investigated by batch method.

The desorption study was carried out by using three kinds of acid solution (0.01 M H_2_SO_4_, HNO_3_, and HCl). 10 mg Fe_3_O_4_@SiO_2_@UiO-66-TPP NPs were shaken with 25 mL uranium solutions at pH 5.0 under ambient temperature (298 K) for 1 h. Under an external magnetic field, uranium loaded Fe_3_O_4_@SiO_2_@UiO-66-TPP NPs were separated and washed with water. Finally, the U(VI) captured by Fe_3_O_4_@SiO_2_@UiO-66-TPP NPs was released by the above-mentioned acid solutions (25 mL) at 298 K for 1 h. The nanoparticles were separated, and the U(VI) was analyzed by ICP-MS.

## Results and discussions

### Characterization of nanoparticles

**Figure 1:**
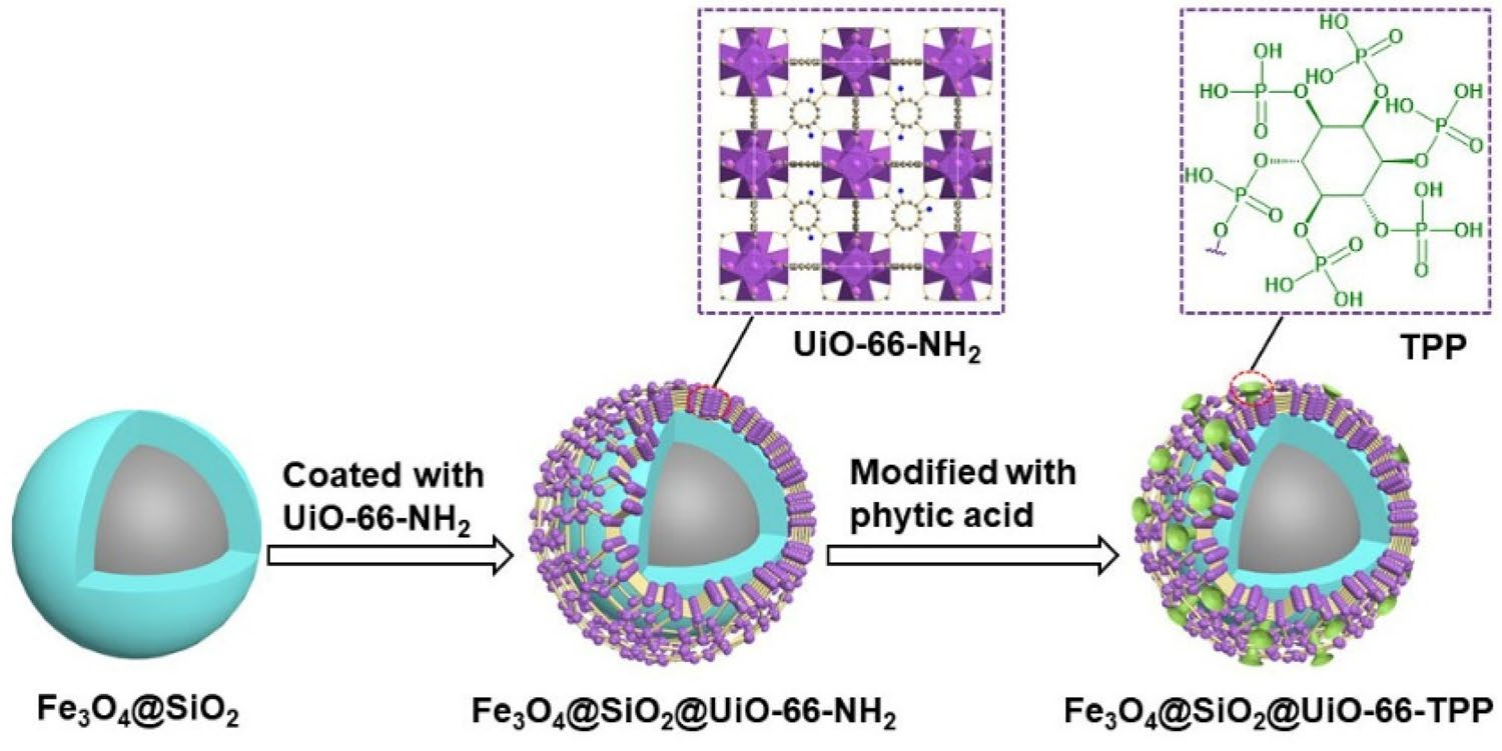
Schematic illustration of the fabrication of Fe_3_O_4_@SiO_2_@UiO-66-TPP NPs.

The assembly process of phosphate group functionalized magnetic metal–organic frameworks nanoparticles (Fe_3_O_4_@SiO_2_@UiO_2_-66-TPP) is illustrated in Fig. 1. Firstly, magnetic iron oxide nanoparticles are coated with a silica layer via sol–gel emulsion method. The surface hydroxyl groups coordinate with Zr^4+^, which assists UiO_2_-66-NH_2_ to settle on the surface of the nanoparticles. Finally, the nanoparticles were dotted with phytic acid through intermolecular hydrogen bonding. To investigate whether Fe_3_O_4_@SiO_2_@UiO_2_-66-TPP shown in Fig. 1 proceed successfully and the properties of the nanoparticles, TEM and composition mapping images, FT-IR, N_2_ adsorption–desorption, VSM and Zeta potential were analyzed in this work.

**Figure 2:**
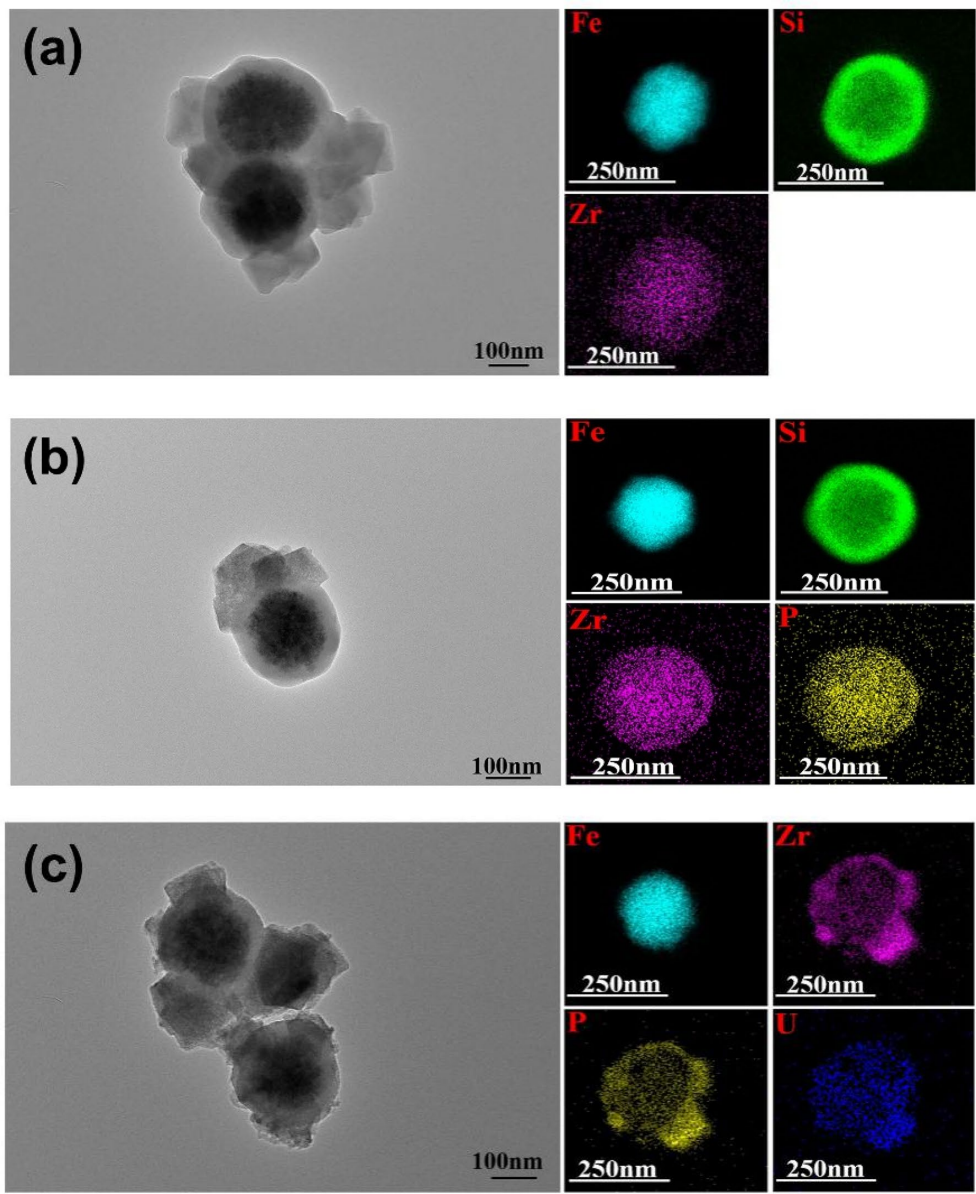
TEM and composition mapping imagesof Fe_3_O_4_@SiO_2_@UiO_2_-66-NH_2_ (**a**), Fe_3_O_4_@SiO_2_@UiO_2_-66-TPP (**b**) NPs before and after (**c**) U(VI) sorption.

The size and morphology of the as-prepared nanoparticles were examined by TEM. As shown in Fig. 2a, the diameter of magnetic iron oxide was 150–270 nm, which was wrapped by a 30-nm-thick silicon layer. The SiO_2_ layer was coated with MOF (UiO-66-NH_2_) layer. Zr element are uniformly dispersed on the nanoparticles. After the modification of phytic acid, the phosphate group was successfully modified on the surface of the material (Fig. 2b). In addition, Fig. 2c showed that P, Zr, and U elements are evenly distributed on nanoparticles after the sorption process, which indicated that U(VI) was successfully captured by Fe_3_O_4_@SiO_2_@UiO_2_-66-TPP NPs and the structure of the nanoadsorbent remained stable.

**Figure 3:**
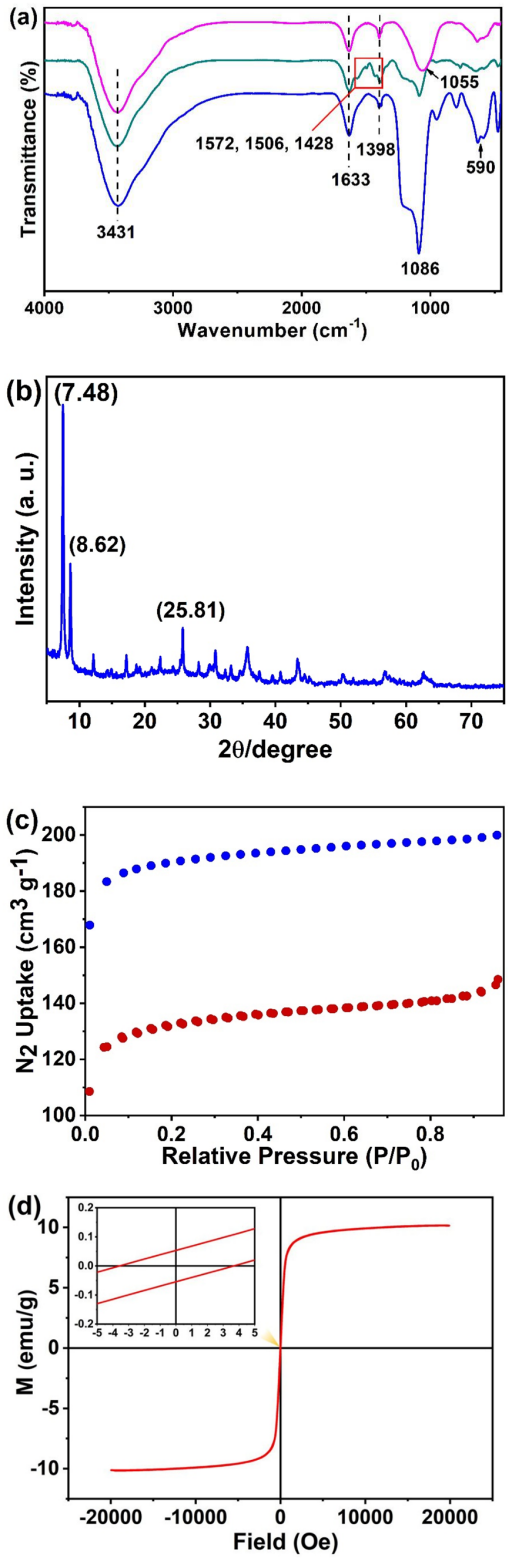
(**a**) FT-IR spectra of Fe_3_O_4_@SiO_2_ (blue line), Fe_3_O_4_@SiO_2_@UiO-66 (green line) and Fe_3_O_4_@SiO_2_@UiO-66-TPP (pink line) NPs. (**b**) XRD pattern of Fe_3_O_4_@SiO_2_@UiO-66-TPP NPs. (**c**) N_2_ adsorption/desorption isotherms of Fe_3_O_4_@SiO_2_@UiO-66-NH_2_ and Fe_3_O_4_@SiO_2_@UiO-66-TPP NPs. (**d**) Magnetization curve of Fe_3_O_4_@SiO_2_@UiO-66-TPP NPs.

The FT-IR spectra of obtained nanoparticles (Fe_3_O_4_@SiO_2_, Fe_3_O_4_@SiO_2_@UiO-66-NH_2_ and Fe_3_O_4_@SiO_2_@UiO-66-TPP) were shown in Fig. 3a. In the spectrum of Fe_3_O_4_@SiO_2_, the peak located at 590 cm^−1^ was assigned to the stretching vibration of Fe–O bond, the peak at 1086 cm^−1^ was ascribed to the Si–O–Si vibration, and the peak at 1628 cm^−1^ and the broad peak centered at 3431 cm^−1^ were ascribed to the stretching vibration of C=O bond and the stretching vibration of O–H and/or N–H bond, respectively. Compared to the above spectrum, there are some new peaks in the spectrum of Fe_3_O_4_@SiO_2_@UiO-66-NH_2_. The additional peak at 1428 cm^−1^ was assigned to the symmetrical stretching vibration of C–O bond, the peaked at 1506 cm^−1^ was assigned to the stretching vibration of C=C bond, and the peak at 1572 cm^−1^ was assigned to the deformation vibration of N–H bond. These bonds all come from the organic monomer (2-aminpterephthalic acid) that constructed the MOF layer. In the spectrum of Fe_3_O_4_@SiO_2_@UiO-66-TPP, the peak at 1055 cm^−1^ was ascribed to the stretching vibration of P=O bond.

The powder XRD pattern of Fe_3_O_4_@SiO_2_@UiO-66-TPP NPs is given in Fig. 3b. UiO-66-NH_2_ was assembled sucessfully on the surface of Fe_3_O_4_@SiO_2_ with characteristic peaks of UiO-66-NH_2_ (2θ = 7.48, 8.62 and 25.81). The crystal size of the lattice calculated from the XRD pattern by Debye–Scherrer equation was about 39.59 nm.

The N_2_ adsorption–desorption isotherms were employed to characterize the specific surface areas of Fe_3_O_4_@SiO_2_@UiO_2_-66-NH_2_ and Fe_3_O_4_@SiO_2_@UiO_2_-66-TPP (Fig. 3c). The N_2_ sorption isotherms showed typical type I curves, suggesting a mesoporous structure. The BET surface areas of the as-synthesized nanoparticles were calculated to be 515.3 and 600.9 m^2^/g, respectively. On account of the presence of the Fe_3_O_4_@SiO_2_ core, the BET surface areas of the as-synthesized nanoparticles is lower than that for UiO-66-NH_2_. The high specific area of Fe_3_O_4_@SiO_2_@UiO_2_-66-TPP NPs was beneficial for the effective removal of U(VI) from solution.

The magnetic property of Fe_3_O_4_@SiO_2_@UiO_2_-66-TPP NPs was characterized by VSM. As shown in Fig. 3d, the nanoparticles were superparamagnetic, the saturation magnetization (*M*_s_) value was 10.15 emu g^−1^, and the magnetic coercivity (Hc) value was 3.67 Oe (Fig. 3 inset). The Fe_3_O_4_@SiO_2_@UiO_2_-66-TPP NPs can be separated and redispersed effectively in aqueous solution with/without an external magnetic field, which is beneficial to its application in U(VI) removal from aqueous solution.

**Figure 4:**
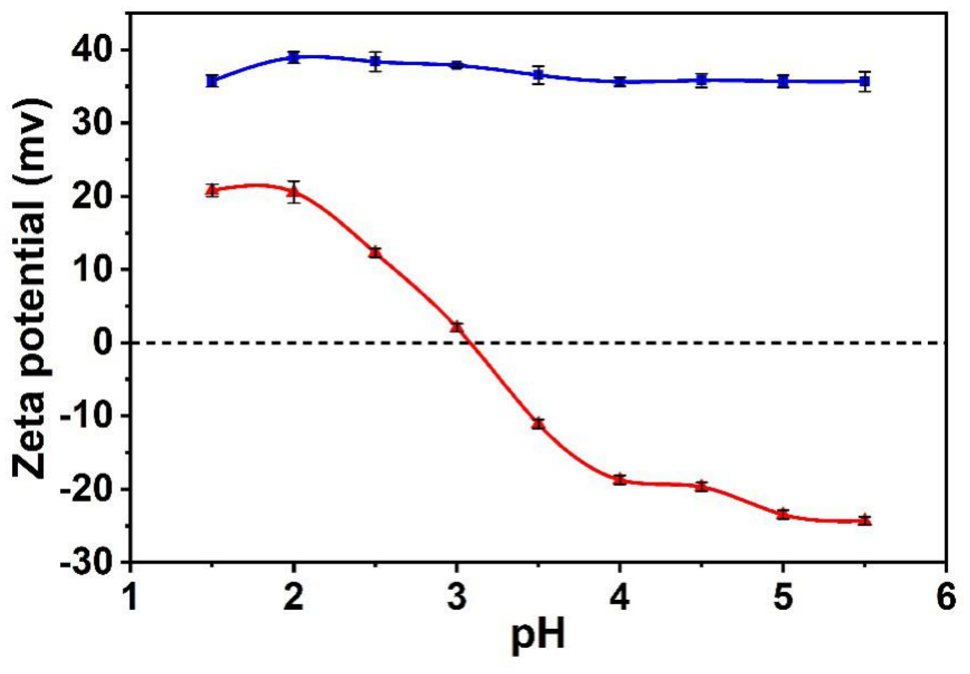
Zeta potential of Fe_3_O_4_@SiO_2_@UiO-66 (blue line) and Fe_3_O_4_@SiO_2_@UiO-66-TPP (red line) NPs.

The surface charge properties of Fe_3_O_4_@SiO_2_@UiO-66-NH_2_ and Fe_3_O_4_@SiO_2_@UiO-66-TPP NPs were evaluated through the measurement of Zeta potentials over pH range 1.5–5.5. As shown in Fig. 4, the surface charges of Fe_3_O_4_@SiO_2_@UiO-66-NH_2_ NPs changed little over a large pH region, while the surface charge of Fe_3_O_4_@SiO_2_@UiO-66-TPP NPs changed greatly, which is due to the decoration of phosphate groups on the nanoparticles. The isoelectric point for Fe_3_O_4_@SiO_2_@UiO-66-TPP NPs was about 3.1. When the pH value was higher than 3.1, the surface of the nanoparticles was negative, and the Zeta potential was − 23.3 mV at the pH 5.0. The high negative charge is beneficial for dispersion of Fe_3_O_4_@SiO_2_@UiO-66-TPP NPs, and diffusion of U(VI) ions towards the surface of the magnetic adsorbents.

### Effect of solution pH

**Figure 5:**
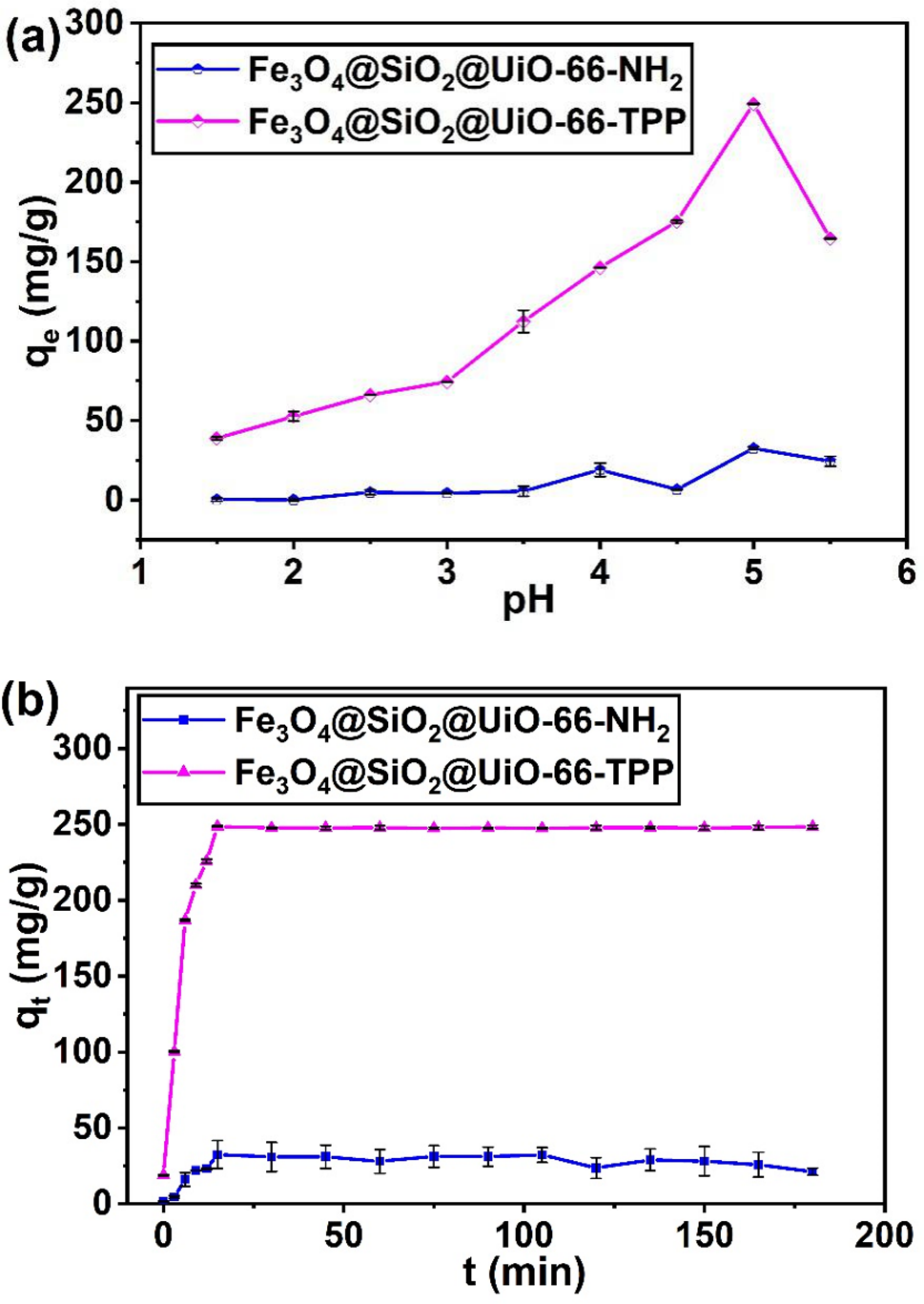
Effects of initial solution pH and contact time (pH 5.0 ± 0.1) on adsorption of U(VI) by Fe_3_O_4_@SiO_2_@UiO_2_-66-NH_2_ and Fe_3_O_4_@SiO_2_@UiO_2_-66-TPP NPs (C_0_ = 100 mg/L, m/V = 0.4 g/L and T = 298 K).

The solution pH is an important parameter that affects the adsorption performance of adsorbents towards U(VI), because it closely related to the speciation of U(VI), the surface charges and binding sites of adsorbents. Herein, the sorption studies of U(VI) on magnetic adsorbents (Fe_3_O_4_@SiO_2_@UiO-66-NH_2_,Fe_3_O_4_@SiO_2_@UiO-66-TPP) were carried out over pH range 1.5–5.5. As shown in Fig. 5a, the adsorption capacity of Fe_3_O_4_@SiO_2_@UiO-66-TPP NPs increased rapidly with increasing pH value up to 5.0 for U(VI), followed by a decline at pH 5.5. The Fe_3_O_4_@SiO_2_@UiO-66-TPP NPs reached maximum adsorption capacity at pH 5.0, and the q_e_ values was 247.7 mg/g. Meanwhile, the adsorption capacity of Fe_3_O_4_@SiO_2_@UiO-66-NH_2_ for U(VI) had no obvious change with the increase of pH, and the maximum adsorption capacity was 32.51 mg/g at pH 5.0.

Uranium mainly exists in the form of UO_2_^2+^ in aqueous solution at or below pH 4.0^68^. When pH < 3.1, the phosphate groups of Fe_3_O_4_@SiO_2_@UiO-66-TPP NPs were positive, and intense competition between H^+^ and UO_2_^2+^ for bindng sites resulted in lower adsorption capacity of the adsorbent. As pH increased, the deprotonation of phosphate groups was promoted, and U(VI) still existed in the form of positive ions, therefore the high and rapid adsorption efficiency could be attributed to the strong electrostatic interaction and chelation between phosphate groups and U(VI). At pH 5.5, the pH environment of solution is not conducive to the chelation between phosphate groups and U(VI), which resulted in the decrease of the adsorption efficiency of the adsorbent for U(VI). When pH is 6, schoepite precipitation (UO_3_·2H_2_O) appeared in the solution, so further adsorption experiments were conducted at pH 5.0.

### Effect of contact time and kinetic studies

Figure 5b showed the influence of contact time on the sorption of U(VI) onto magnetic adsorbents (Fe_3_O_4_@SiO_2_@UiO-66, Fe_3_O_4_@SiO_2_@UiO-66-TPP). The adsorption of Fe_3_O_4_@SiO_2_@UiO-66-TPP towards U(VI) quickly reached equilibrium within 15 min, which is due to the strong complexation of phosphate group on the Fe_3_O_4_@SiO_2_@UiO-66-TPP with U(VI).

**Figure 6:**
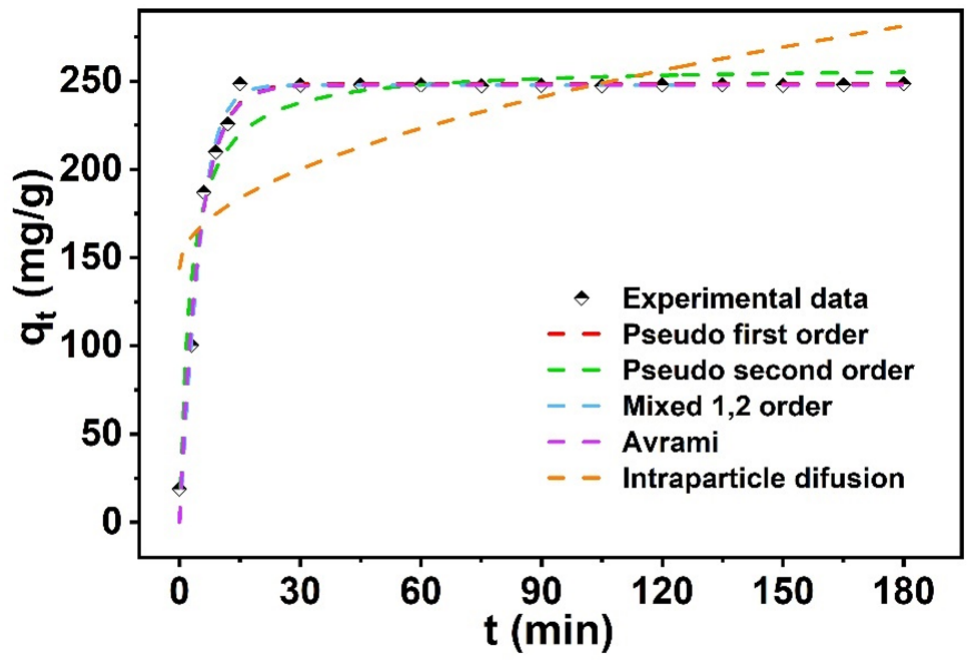
Adsorption kinetic models (C_0_ = 100 mg/L, m/V = 0.4 g/L, pH = 5.0 ± 0.1, and T = 298 K).

The pseudo-first-order, pseudo-second-order, mixed 1,2 order, intraparticle diffusion and Avrami kinetic models were used to further explore the interaction mechanism (Fig. 6).

Pseudo-first-order equation:

4 $$q_{t} = q_{e} \left( {1 - e^{{ - k_{1} t}} } \right)$$

Pseudo-second-order equation:

5 $$q_{t} = \frac{{q_{e}^{2} k_{2} t}}{{1 + q_{e} k_{2} t}}$$

Mixed 1,2 order equation:

6 $$q_{t} = q_{e} \frac{{1 - exp\left( { - kt} \right)}}{{1 - f_{2} \;exp\left( { - kt} \right)}}$$

Intraparticle diffusion equation:

7 $$q_{t} = k_{ip} \sqrt t + c_{ip}$$

Avrami equation:

8 $$q_{t} = q_{e} \left[ {1 - exp\left( { - k_{av} t} \right)^{{n_{av} }} } \right]$$

where q_e_ and q_t_ (mg/g) are the sorption amounts of U(VI) on Fe_3_O_4_@SiO_2_@UiO-66-TPP at equilibrium and at time t (min), respectively; k_1_ (1/min) and k_2_ (1/min) represent the rate constants of the pseudo-first-order model and pseudo-second-order model, respectively; k (mg/(g min)) is the adsorption rate constant, f_2_ (dimensionless) and K_ip_ (mg/(g min^0.5^)) are the coefficient of mixed 1,2 order and intraparticle diffusion, respectively; c_ip_ (mg/g) is the intraparticle diffusion constant; k_av_ (1/min) is the Avrami rate constant; n_av_ (dimensionless) is the Avrami component.

Table 1 listed the fitting parameters calculated from these kinetic models (Fig. 6). The pseudo-first order, pseudo-second order, mixed 1, 2-order and Avrami models with a high correlation coefficient (R^2^ > 0.98) described the adsorption process of U(VI) better than the intrsparticle difusion kinetic model (R^2^ = 0.44).

**Table 1:** Kinetic model constants for the adsorption of U(VI) onto Fe_3_O_4_@SiO_2_@UiO_2_-66-TPP NPs.

Kinetic models	Parameters	Fe_3_O_4_@SiO_2_@UiO-66-TPP
Pseudo first order	K_1_	0.206
	q_e_	248.72
	R_2_	0.987
Pseudo second order	K_2_	0.00145
	q_e_	258.83
	R_2_	0.947
Mixed 1, 2 order	K	0.290
	q_e_	247.760
	f_2_	− 0.889
	R^2^	0.989
Avrami	q_e_	247.67
	k_av_	1.0
	n_av_	0.209
	R^2^	0.987
Intraparticle difusion	K_ip_	10.217
	C_ip_	144.136
	R^2^	0.439

### Effect of initial U(VI) concentration and isotherm studies

**Figure 7:**
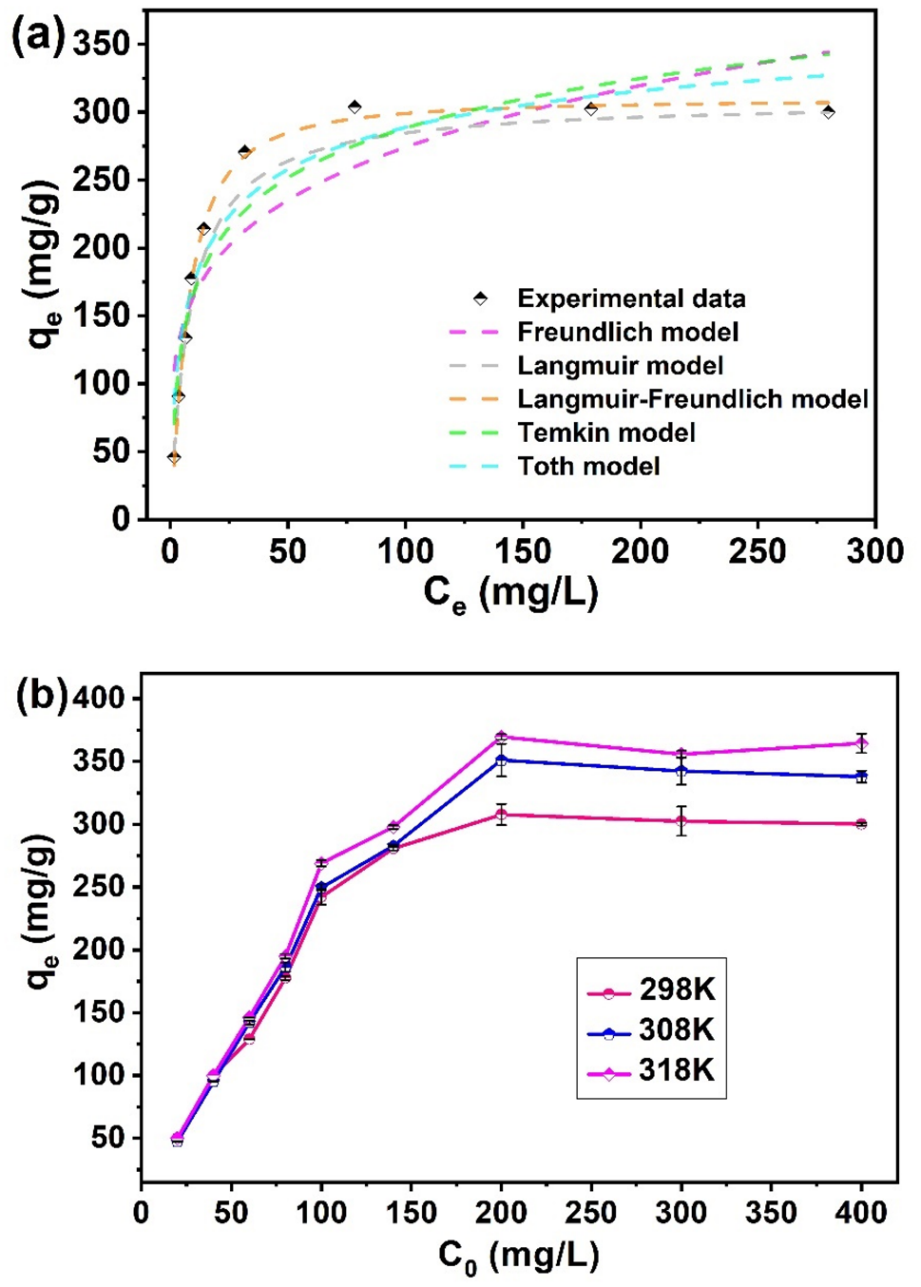
(**a**) Adsorption isotherm models of U(VI) on Fe_3_O_4_@SiO_2_@UiO_2_-66-TPP NPs (pH = 5.0 ± 0.1, T = 298 K and t = 60 min). (**b**) Effects of temperature on adsorption capacity of the adsorbent towards U(VI).

The adsorption behavior of U(VI) on Fe_3_O_4_@SiO_2_@UiO-66-TPP NPs was investigated by changing the initial U(VI) concentration from 1.6 to 280 mg/L. As shown in Fig. 7a, the adsorption amounts increased quickly with the enhancement of the initial U(VI) concentration from 1.6 to 78.5 mg/L. Then the adsorption reached the maximum capacity when the initial U(VI) concentration was above 200 mg/L, which is ascribed to the saturation of phosphate group binding sites embellished on Fe_3_O_4_@SiO_2_@UiO-66-TPP NPs. The most widespread isotherm models (Langmuir, Freundlich, Temkin, Sips, Toth and Langmuir–Freundlich,) were applied to describe the adsorption behavior (Fig. 7a).

Langmuir isotherm equation:

9 $$q_{e} = \frac{{q_{m} K_{L} C_{e} }}{{1 + K_{L} C_{e} }}$$

Freundlich isotherm equation:

10 $$q_{e} = K_{F} \times C_{e}^{{{\raise0.7ex\hbox{$1$} \!\mathord{\left/ {\vphantom {1 n}}\right.\kern-\nulldelimiterspace} \!\lower0.7ex\hbox{$n$}}}}$$

Temkin isotherm equation:

11 $$q_{e} = \frac{RT}{{b_{T} }}In\left( {A_{T} C_{e} } \right)$$

Toth isotherm equation:

12 $$q_{e} = \frac{{K_{e} C_{e} }}{{\left[ {1 + (K_{L} C_{e} )^{n} } \right]^{1/n} }}$$

Langmuir–Freundlich isotherm equation:

13 $$q_{e} = \frac{{q_{MLF} (K_{LF} C_{e} )^{MLF} }}{{1 + (K_{LF} C_{e} )^{MLF} }}$$

where q_e_ (mg/g) is the equilibrium adsorption capacity (mg/g), q_m_ and q_MLF_ (mg/g) are the maximum adsorption capacities, K_L_ is the adsorption equilibrium constant (L/mg), K_F_ (mg/g)/(mg/L) is the Freundlich parameter, n is the Freundlich intensity parameter, C_e_ (mg/L) is the equilibrium concentration, b_T_ and A_T_ (L/g) represent the constant and equiliburium constant of Temkin isotherm model, respectively, R (8.314 J/(mol K)) is the universal gas constant, T is the absolute temperature at 298 K, n and K_LF_ are the isotherm constants, MLF represent the heterogeneous parameter.

The Langmuir and Freundlich isotherm models describes a monolayer and multilayer adsorption of targeted molecules on adsrobent surfaces^69–71^. Temkin model incorporates a linear variation of the adsorption enthalpy, which is an extension of Langmuir model^72^. Toth model is also an extension of traditional Langmuir model, which takes into account of heterogeneity and non-uniformity of the binding sites on the adsorbent surface^73^. Langmuir–Freundlich model represents a combination of Langmuir and Freundlich isotherm models, at low concentration it reduces to Freundlich isotherm, it predicts a Langmuir monolayer adsorption^74^. The model isotherm parameters obtained from fitting curves (Fig. 7a) were summarized in Table 2. Obviously, the adsorption process followed Langmuir–Freundlich and Langmuir isotherm models due to high correlation coefficients (R^2^). The maximum adsorption capacities calculated from Langmuir–Freundlich and Langmuir isotherm models were very close (310.1 and 308.9 mg/g, respectively) to practical adsorption amount (307.8 mg/g).

**Table 2:** Adsorption isotherm model constants derived from Langmuir and Freundlich isotherms.

Isotherm models	Parameters	Fe_3_O_4_@SiO_2_@UiO-66-TPP
Langmuir	q_m_	308.9
	K_L_	0.118
	R_L_	0.0782
	R^2^	0.991
Freundlich	K_f_	99.036
	1/n	4.522
	R^2^	0.778
Temkin	b_T_	47.010
	A_T_	2.376
	R^2^	0.895
Toth	K_e_	308.0
	K_L_	0.731
	n	0.414
	R^2^	0.909
Langmuir–Freundlich	q_MLF_	310.1
	K_LF_	0.136
	MLF	1.265
	R^2^	0.996

To investigate the effect of temperature on the U(VI) adsorption capacity of the Fe_3_O_4_@SiO_2_@UiO-66-TPP NPs, the adsorption isotherms of U(VI) on Fe_3_O_4_@SiO_2_@UiO-66-TPP NPs were conducted at three temperatures (298, 313, 328 K) at pH 5.0 (Fig. 7b). The thermodynamic parameters, including ∆S^0^ (entropy change), ∆H^0^ (enthalpy change) and ∆G^0^ (Gibbs free energy change), were calculated by Van’t Hoff equation and Gibb’s free energy function, to reveal whether the adsorption process was endothermic and exothermic, spontaneous or nonspontaneous.

Van’t Hoff equation:

14 $${\text{InK}}_{{\text{d}}} = \frac{{\Delta {\text{S}}^{0} }}{{\text{R}}} - \frac{{\Delta {\text{H}}^{0} }}{{{\text{RT}}}}$$

Gibb’s free energy function:

15 $$\Delta {\text{G}}^{0} = \Delta {\text{H}}^{0} - {\text{T}}\Delta {\text{S}}^{0}$$

where K_d_ is the equilibrium constant at different temperature, R is the gas constant (8.314 J/(mol K)), ∆S^0^ (J (mol K)), ∆H^0^ (kJ/mol), ∆G^0^ (kJ/mol) are the entropy, enthalpy and Gibbs free energy change, respectively.

The thermodynamic parameters were listed in Table 3. The positive ∆H^0^ value proved that the adsorption of U(VI) on Fe_3_O_4_@SiO_2_@UiO-66-TPP was endothermic. The positive ∆S^0^ value revealed an increase in the randomness at the solid–liquid interface during the adsorption process. The negative ∆G^0^ value indicated that the adsorption process was spontaneous and Fe_3_O_4_@SiO_2_@UiO-66-TPP owned high affinity toward U(VI) in aqueous solution. ∆G^0^ values decreased with temperature increasing, indicating that higher temperature, the higher the spontaneous trend of spontaneous adsorption of U(VI) on Fe_3_O_4_@SiO_2_@UiO-66-TPP.

**Table 3:** Thermodynamic parameters for the U(VI) adsorption onto Fe_3_O_4_@SiO_2_@UiO-66-TPP NPs.

$\Delta {\text{H}}^{{0}} \left( {\text{kJ/mol}} \right)$	$\Delta {\text{S}}^{{0}} \left[ {{\text{J/(mol}}\;{\text{K)}}} \right]$	$\Delta {\text{G}}^{{0}} \left( {\text{kJ/mol}} \right)$		
		298 K	308 K	313 K
35.64	172.89	− 15.87	− 17.61	− 19.33

### Effect of ionic strength and coexisting ions

**Figure 8:**
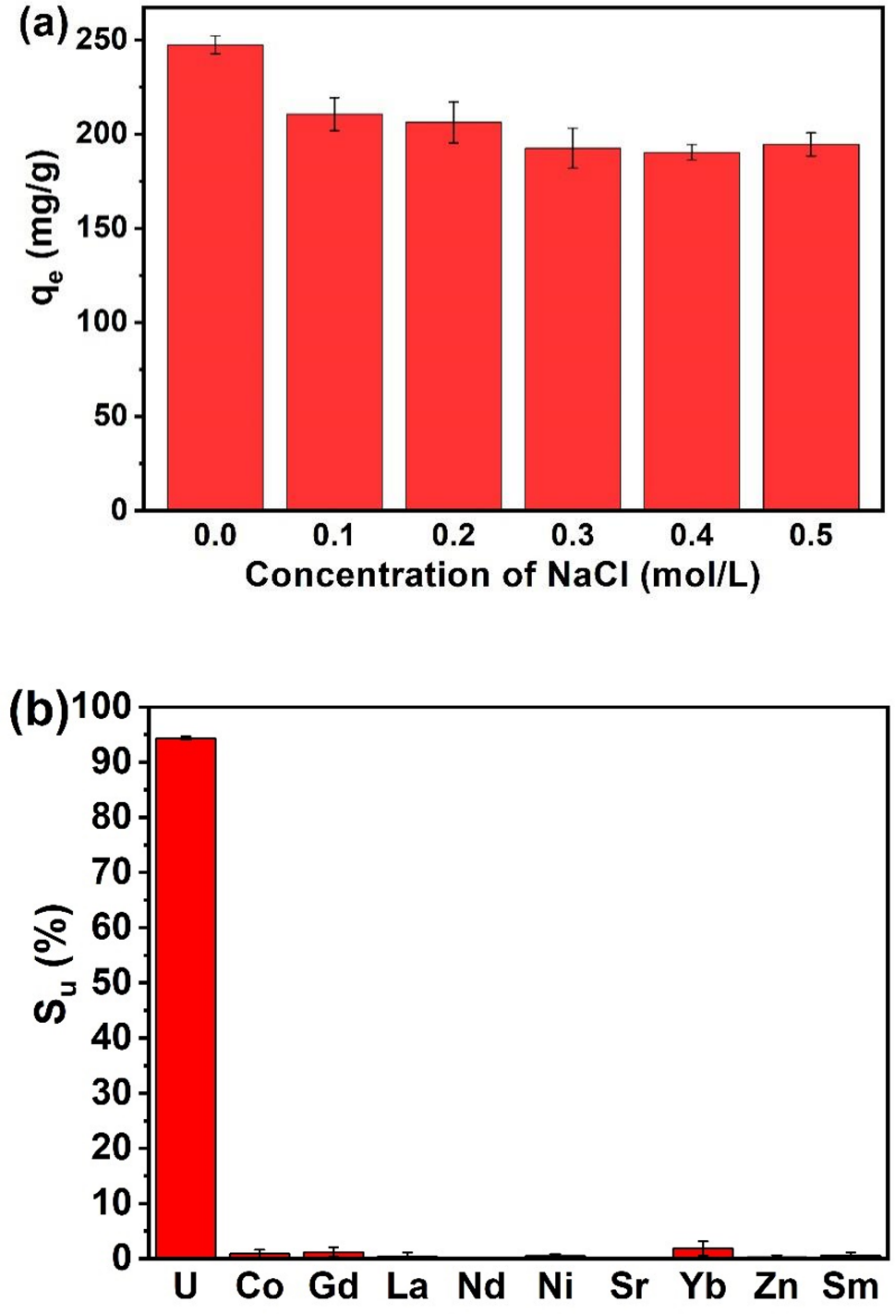
(**a**) Effect of ionic strength on removal of U(VI) by Fe_3_O_4_@SiO_2_@UiO_2_-66-TPP NPs (C_0_ = 100 mg/L, m/V = 0.4 g/L, pH = 5.0 ± 0.1, and T = 298 K). (**b**) Effect of competitive ions on the selective sorption of U(VI) onto the sorbent (C_0_ = 0.5 mmol/L for all ions, pH = 5.0 ± 0.1, T = 298 K, V = 25 mL, t = 60 min, and m = 10 mg).

Nuclear industrial wastewater and seawater contain many kinds of ions, which may affect the mutual interaction between Fe_3_O_4_@SiO_2_@UiO-66-TPP and U(VI). Therefore, the effects of ionic strength and coexisting ions on the adsorption selectivity and capacity of Fe_3_O_4_@SiO_2_@UiO-66-TPP for U(VI) should be further investigated. Figure 8 shows the influence of ionic strength on the adsorption capacity of Fe_3_O_4_@SiO_2_@UiO-66-TPP. As shown in Fig. 8a, the adsorption capacity decreased slightly by about 18% in the NaCl concentration range of 0.1–0.5 mol/L, which may be attributed to the decrease of ion transfer rate and the interference of electrostatic interaction caused by higher NaCl concentration.

As shown in Fig. 8b, the adsorption capacity of Fe_3_O_4_@SiO_2_@UiO-66-TPP for U(VI) is significantly higher than that of other coexisting ions. The uranium-selectivity (*S*_u_) of Fe_3_O_4_@SiO_2_@UiO-66-TPP was 94.4% calculated according to the Eq. (3), exhibiting an excellent selectivity of the magnetic adsorbent for U(VI). The mutual interaction of Fe_3_O_4_@SiO_2_@UiO-66-TPP and U(VI) depends on the chelation of U(VI) with phosphate groups grafted on the magnetic adsorbent. Phosphate group is more inclinded to coordinate with actinides than other metal ions in aqueous solutions, leading to the excellent selectivity of Fe_3_O_4_@SiO_2_@UiO-66-TPP for U(VI). Meanwhile, the phosphate group endowed Fe_3_O_4_@SiO_2_@UiO-66-TPP with high adsorption capacity (320.3 mg/g) and fast adsorption rate (15 min). The adsorption selectivity, capacity and rate of Fe_3_O_4_@SiO_2_@UiO-66-TPP are higher than other magnetic adsorbents listed in Table 4.

**Table 4:** Comparison of the maximum adsorption capacity of Fe_3_O_4_@SiO_2_@UiO-66-TPP NPs with other magnetic adsorbents.

Adsorbents	q_max_ (mg/g)	time	*S*_u_ (%)	pH	References
UiO-66	109.9	4 h	Not analyzed	5.5	^75^
UiO-66-NH_2_	114.9	4 h	Not analyzed	5.5	^75^
Fe_3_O_4_/P(GMA-AA-MMA)	< 200	Not analyzed	37	4.5	^76^
M/SiO_2_-Si-SBC	114.7	10 h	Not analyzed	5.0	^77^
SβCD-APTES@Fe_2_O_3_	286	3 h	Not analyzed	6.0	^78^
MNHA	310	2 h	Not analyzed	5.0	^79^
Fe_3_O_4_@C@Ni–Al LDH	227	3 h	Not analyzed	6.0	^80^
AO-Fe_3_O_4_/P(GMA-AA-MMA)	255.0	30 min	57	4.5	^76^
Fe_3_O_4_/P(AA-MMA-DVP)	413.2	45 min	95.8	4.5	^81^
Fe_3_O_4_/P(GMA-AA-MMA)	274.7	20 min	77	4.5	^82^
Fe_3_O_4_@AMCA-MIL53(Al)	227.3	1.5 h	Not analyzed	5.5	^83^
Fe_3_O_4_@MnO_X_	106.7	120 min	Not analyzed	5.0	^84^
Fe_3_O_4_@SiO_2_@UiO-66-NH_2_	27.7	130 min	Not analyzed	5.0	This work
Fe_3_O_4_@SiO_2_@UiO-66-TPP	307.8	15 min	94.4	5.0	

### Desorption analysis

**Figure 9:**
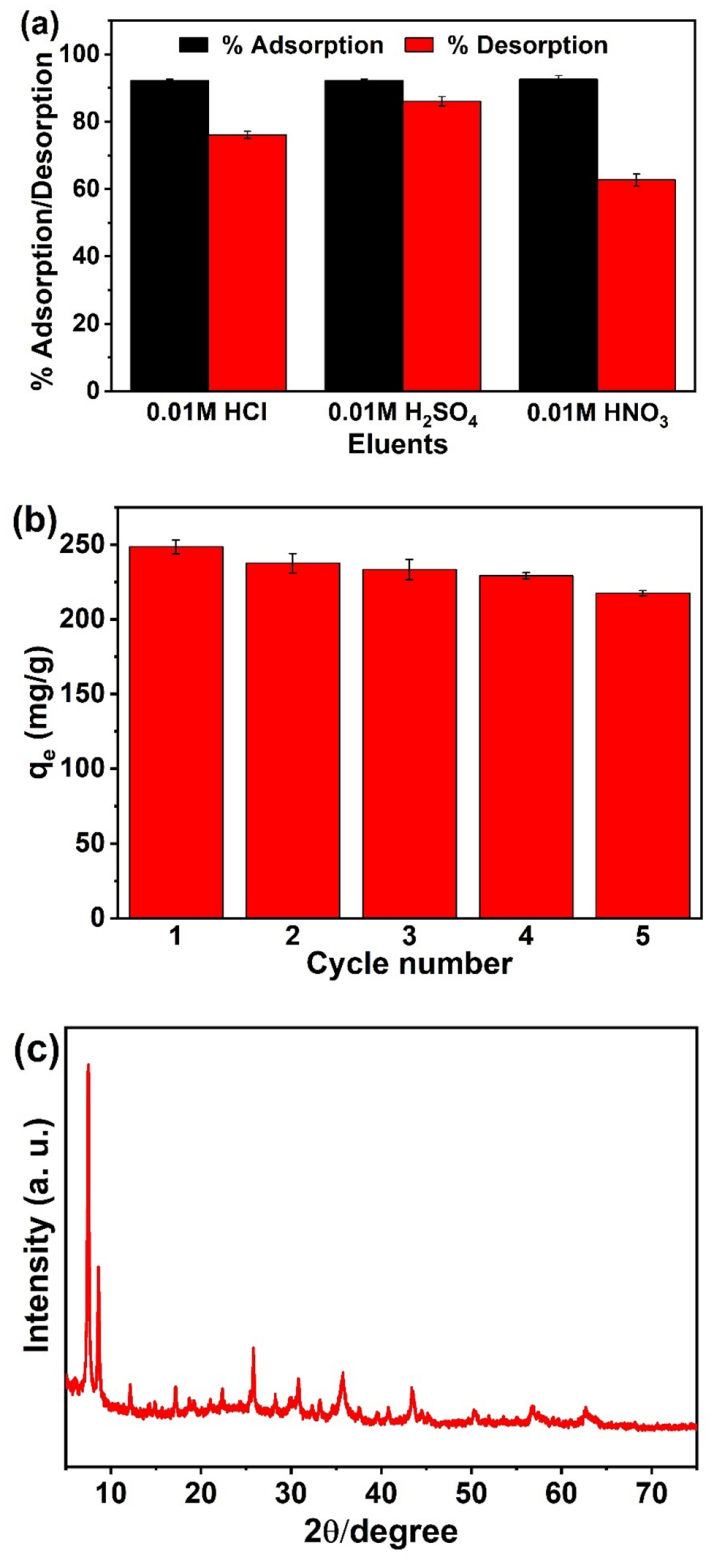
Desorption (**a**) and recyclability (**b**) studies of Fe_3_O_4_@SiO_2_@UiO_2_-66-TPP NPs. (**c**) XRD pattern of the utilized Fe_3_O_4_@SiO_2_@UiO_2_-66-TPP NPs after 5 cycles.

The selection of suitable eluent for desorption of U(VI) from the uranium-loaded Fe_3_O_4_@SiO_2_@UiO-66-TPP NPs may provide better recovery of U(VI). The desorption of U(VI) from Fe_3_O_4_@SiO_2_@UiO-66-TPP NPs was studied in a batch mode using different eluents (HCl, H_2_SO_4_, HNO_3_) at a concentration of 0.01 M. As shown in Fig. 9a, H_2_SO_4_ was the best eluent with a elution rate of 86.0% due to the extensive protonation on the adsorbent surface, which was further used for five successive adsorption–desorption cycles under identical experimental conditions. As shown in Fig. 9b, the adsorption capacity of U(VI) on Fe_3_O_4_@SiO_2_@UiO-66-TPP NPs decreased from 248.5 to 217.5 mg/g after five regeneration, which was approximately 12.5% reduction. Moreover, the XRD analysis result indicated that the utilized Fe_3_O_4_@SiO_2_@UiO-66-TPP NPs retained the crystal structure stability after 5 cycles (Fig. 9c). These results indicate the potential reusability of Fe_3_O_4_@SiO_2_@UiO-66-TPP NPs for U(VI) removal from aqueous medium.

### Application in adsorption of uranium from pre-treated seawater

**Figure 10:**
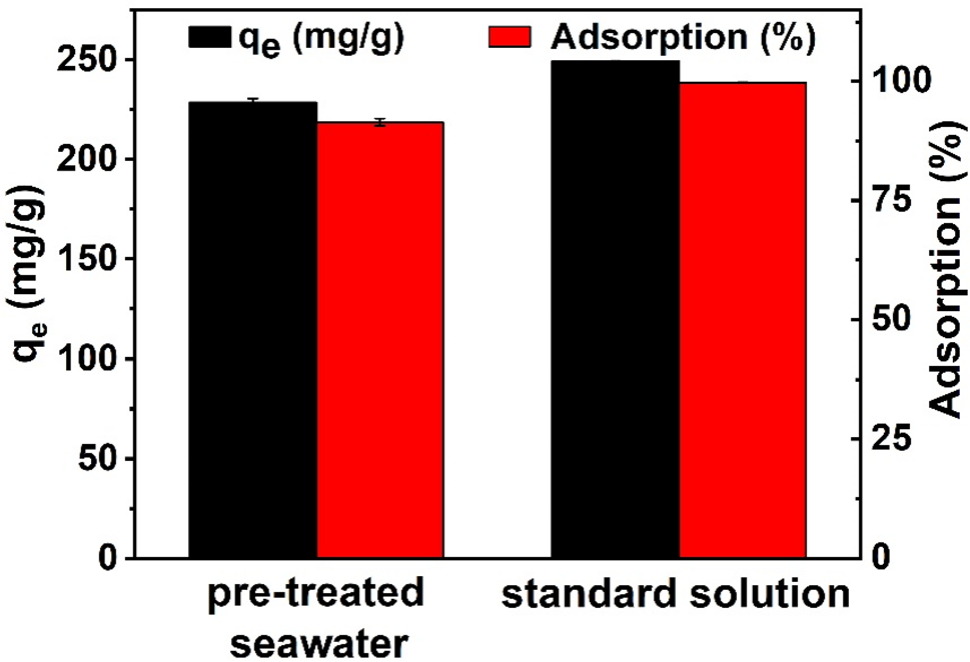
Application in adsorption of U(VI) from pre-treated seawater.

From the experimental results obtained, it can be seen that Fe_3_O_4_@SiO_2_@UiO-66-TPP NPs has a high adsorption rate and a high adsorption capacity for U(VI) and the adsorption can reach equilibrium in a very short time. Furthermore, the adsorbent exhibits a very good selectivity for uranium ions in the presence of coexisting ions. In order to evaluate the potential application of Fe_3_O_4_@SiO_2_@UiO-66-TPP NPs for U(VI) removal from seawater, we carried out the experiments on adsorption of U(VI) from the uranium-doped seawater. The natural seawater used in the adsorption experiments came from near-surface seawater from Tianjin, China. The concentrations of uranium ions in the uranium-doped seawater is 100 mg/L, and the pH of the solution was adjusted to 5.0. The adsorption capacity of U(VI) on Fe_3_O_4_@SiO_2_@UiO-66-TPP NPs from the pre-treated seawater was 228.6 mg/g, and the adsorption rates reached 91.4%, which were only a little bit lower than the adsorption capacity (249.3 mg/g) and adsorption efficiency (99.7%) from ultrapure water (Fig. 10). This demonstrates that Fe_3_O_4_@SiO_2_@UiO-66-TPP NPs have great potential application in the removal of U(VI) from radionuclide-polluted seawater.

## Conclusions

In summary, a novel phosphate group functionalized magnetic metal–organic framework nanocomposite composed of magnetic Fe_3_O_4_ NPs and UiO-66-NH_2_ was successfully prepared and characterized by various techniques. The magnetic nanocomposite was used to remove U(VI) from aqueous solution. The nanocomposite was interspersed with phosphate group that forms a stable chelate with U(VI), and the adsorption of Fe_3_O_4_@SiO_2_@UiO-66-TPP NPs for U(VI) reached equilibrium in 15 min, the maximum adsorption capacity is 307.8 mg/g, and the selectivity (*S*_u_) is 94.4% in aqueous media including 10 coexisting ions. Fe_3_O_4_@SiO_2_@UiO-66-TPP NPs possess high adsorption capacities, outstanding selectivity and excellent recylability towards U(VI), which were endowed with magnetic separation performance by Fe_3_O_4_ cores, but the low adsorption efficiency at pH < 3.1 under anoxic condition limited its practical applications. Neverthless, the research work provides a novel strategy to assemble phosphate group-functionalized MOFs.
